# Identifying Temporal Codes in Spontaneously Active Sensory Neurons

**DOI:** 10.1371/journal.pone.0027380

**Published:** 2011-11-08

**Authors:** Alexander B. Neiman, David F. Russell, Michael H. Rowe

**Affiliations:** 1 Neuroscience Program, Ohio University, Athens, Ohio, United States of America; 2 Department of Physics and Astronomy, Ohio University, Athens, Ohio, United States of America; 3 Department of Biological Sciences, Ohio University, Athens, Ohio, United States of America; University of Michigan, United States of America

## Abstract

The manner in which information is encoded in neural signals is a major issue in Neuroscience. A common distinction is between rate codes, where information in neural responses is encoded as the number of spikes within a specified time frame (encoding window), and temporal codes, where the position of spikes within the encoding window carries some or all of the information about the stimulus. One test for the existence of a temporal code in neural responses is to add artificial time jitter to each spike in the response, and then assess whether or not information in the response has been degraded. If so, temporal encoding might be inferred, on the assumption that the jitter is small enough to alter the position, but not the number, of spikes within the encoding window. Here, the effects of artificial jitter on various spike train and information metrics were derived analytically, and this theory was validated using data from afferent neurons of the turtle vestibular and paddlefish electrosensory systems, and from model neurons. We demonstrate that the jitter procedure will degrade information content even when coding is known to be entirely by rate. For this and additional reasons, we conclude that the jitter procedure by itself is not sufficient to establish the presence of a temporal code.

## Introduction

A fundamental question in sensory neuroscience is how information is encoded in spike trains. The question often takes the form of distinguishing between rate codes, in which information is encoded in terms of the number of spikes within an encoding window, and temporal codes, in which the position of spikes within an encoding window carries information beyond that available from the number of spikes in the window [1]. Temporal codes are usually associated with nonlinear relations between the Fourier components of a stimulus and a neuronal response [1], [2], i.e. correlations between a particular frequency component of a stimulus and higher-frequency components of the response. These nonlinear relations provide information about the stimulus beyond that provided by linear correlations within the frequency band of the stimulus. In contrast, rate coding can be nonlinear, but it is characterized by a lack of correlation between Fourier components of the stimulus and higher-frequency components of the response, or by the fact that such nonlinear correlations, when present, do not provide any additional information about the stimulus. The pioneering work of Adrian [3] provided clear evidence that cutaneous sensory afferents use firing rate to encode stimulus intensity (a concise history of this work and related issues is in [4]). More recent work on a number of sensory systems has provided equally compelling evidence that precise spike timing can carry information beyond that available from measures of firing rate (e.g., [5]–[17] among many others).

An additional consideration is that primary afferent neurons in a variety of sensory systems exhibit an ongoing background discharge. Examples include vestibular afferents [18], [19], and electroreceptor afferents in several aquatic species [20]–[22]. Such background firing can arise from a variety of mechanisms including intrinsic oscillators, intrinsic noise, or random synaptic events. The resulting discharges span the spectrum from highly periodic to completely random spike sequences. Several studies have attempted to relate the properties of this background discharge to the stimulus encoding properties of afferents, by stimulating a system with time-varying Gaussian noise, and assessing information transmission based on various information metrics calculated from their responses (reviewed in [4], [10], [23]).

To assess the relative importance of firing rate versus precise spike timing in stimulus encoding, a computational procedure is often used in which the time of each spike is “jittered” by the addition of a variable time offset, chosen randomly from a zero-mean distribution [6], [20], [24]–[26]. The jittering produces a surrogate data set for which information metrics can be computed and compared to the same metrics computed from the original data. If the addition of jitter significantly decreases the information transmission and/or encoding efficiency of the afferent, as happens, for example, for some vestibular afferents [24], then the existence of a temporal encoding scheme is inferred.

However, the distinction between a rate code and a timing code can be problematic for a number of reasons. First, as discussed by Theunissen and Miller [1], the use of spike timing to encode transient or high frequency components of a stimulus can be consistent with a rate coding scheme, e.g. [6], [27]. Nor does the use of a temporal encoding scheme require high spike timing precision. Even in the case of a highly periodic spontaneously firing neuron, which like all self-sustained oscillators is inherently nonlinear, the response magnitude at different points in the neuron's cycle (its phase response curve) can be closely related to its linear response function [28], [29]. Weak stimuli can be linearly encoded in the instantaneous firing rate of a periodically firing neuron, and this encoding can be accounted for within the framework of linear response theory [28], [29]. Thus, the intrinsic timing precision of a periodically firing neuron is not necessarily indicative of a temporal code as understood in the current neuroscience literature.

Second, the linear stimulus reconstruction technique [1], [23], [30] that is typically used in conjunction with the jitter procedure treats a neuron as a *linear* “black box” whose transfer function is tuned to minimize the mean square error of stimulus estimation. This technique essentially assumes a rate code, since the stimulus is estimated by convolving a spike train with the response function of the optimal *linear* filter. Adding external noise in the form of jitter is equivalent to a distortion of the transfer function of the optimal filter. Thus, conclusions about the existence of a nonlinear time code drawn solely from application of a linear stimulus reconstruction technique may be questionable.

Third, the rationale for jitter analysis is based on the assumption that the standard deviation (SD) of the jitter distribution is small relative to the duration of an “encoding window”, so that the number of spikes within the window is unaffected, and only their temporal position within the window is altered. Thus, the SD of the jitter is normally chosen to be much smaller than the characteristic time scale of the stimulus on the assumption that this will be less than the duration of the encoding window. However, since the duration of the encoding window itself is never determined, this assumption cannot be validated, and so the results of artificial jittering should be interpreted with caution.

Here, we develop an analytical framework that provides a detailed, quantitative assessment of the effects of artificial jitter on spike train metrics commonly used to analyze sensory encoding: coefficient of variation, serial correlations, power spectral density, transfer functions, and coherence functions. This theoretical analysis allows us to specify precisely the relationships between these metrics as calculated for original and jittered spike trains. Using this framework, we show that jitter alters the higher order statistics of spike trains by introducing spurious serial correlations among interspike intervals. This can alter encoding properties. More importantly, we show that for weak stimuli and linear responses, jitter merely increases the noise in the background discharge. This occurs independently of any applied lower-frequency stimulus, and with minimal effects on stimulus-response gain. The additional noise from jitter results in suppression of the stimulus-response coherence (or the linear reconstruction kernel), and consequently of the mutual information rate, as estimated with the linear reconstruction technique. We illustrate these theoretical results by applying them to a model neuron with gamma-distributed interspike intervals, to a phase model of a periodically firing neuron, and to experimental data from vestibular and electroreceptor afferents. Although we focus on spontaneously active neurons, the theory, results, and conclusions we develop have broad applicability to analyses of sensory encoding.

## Methods

### Experimental

#### Turtle

The activity of vestibular posterior canal afferents was recorded in *in vitro* preparations of red-eared turtles, *Trachemys (Pseudemys) scripta elegans,* of 10–13 cm carapace length, as in [31]. Turtles were sacrificed by decapitation, and further dissection was done in a bath of oxygenated turtle Ringer's solution. After removal of the dorsal cranium, the brainstem was transected at the meso-thalamic junction, and the rostral portions discarded. A small hole was drilled in the bone overlying the posterior canal, approximately 2–3 mm from the posterior ampulla, to allow placement of a mechanical probe on the posterior semicircular duct. Stimuli consisted of indentations of the posterior semicircular duct using this probe. The head was then placed in a humidified recording chamber that was continuously infused with mixture of 95% O_2_/5% CO_2_. All procedures were approved by the Ohio University Institutional Animal Care and Use Committee (IACUC) (protocol number L01-35). Afferent spikes were recorded with glass micropipettes filled with 2 M NaCl and having electrical impedances of 50–100 MΩ. The electrodes were inserted into the posterior division of the VIIIth nerve along the antero-dorsal margin [32]. Signals from the electrodes were amplified, digitized at 10 kHz, and stored for offline analysis using Spike2™ software (Cambridge Electronic Design, CED).

#### Paddlefish

The spontaneous firing of electroreceptor afferents of paddlefish (*Polyodon spathula*) was recorded in *in vivo* preparations, as in [21]. This passive electrosensory system has cutaneous electroreceptors of the cathodally excited “Lorenzinian” ampullary type, like sharks and rays. Paddlefish are named for their “rostrum”, a flattened sensory appendage shaped like a canoe paddle, covered with electroreceptors, projecting forward from the head. A fish was held in an all-plastic chamber, maintained by a stream of oxygenated 22°C water into the mouth. A special advantage of paddlefish is that water flow or turbulence around electroreceptors on the rostrum, which might disturb spontaneous afferent firing, could be stilled by partitioning the chamber bath using a slab of agarose across the base of the rostrum. The cranium was opened dorsally to expose the sensory ganglion of the anterior lateral line cranial nerve, on one or both sides. A tungsten microelectrode was advanced into this ganglion to record single-unit spikes from an afferent's cell body. If used, electrical stimuli were applied from a 2 mm dipole electrode, or between two large chlorided Ag plates at the chamber ends, connected to a low-noise linear constant-current electrical isolator, commanded by a CED computer interface replaying arbitrary waveforms such as band-limited Gaussian noise. Data acquisition was similar to that for turtle. Data were from experiments at University of Missouri-St. Louis, under an IACUC-approved animal use protocol (W01-13) there.

The duration of Gaussian stimuli was 300–500 s for the turtle posterior canal afferents and 180 s for the paddlefish electroreceptor afferents.

### Data analysis

Data analyses were performed offline using custom software programmed in MATLAB. The same analyses were used for both experimental recordings and numerical simulations. Definitional equations for the analyses are included for clarity.

#### Spontaneous discharge statistics

Three metrics were used to characterize spontaneous discharge: the coefficient of variation of the interspike interval distribution, serial correlation coefficients in ISI sequences, and the power spectral density of a spike train. Given a sequence of spike times *t*_1_,*t*_2_,...,*t_K_*, the corresponding sequence of interspike intervals (ISIs) is 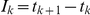. The variability of an ISI distribution was characterized using the coefficient of variation, 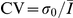, where 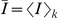 is the mean ISI, 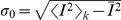 is the SD of the ISI distribution, and 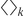 denotes averaging over *k* intervals. The mean firing rate is 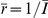.

Serial correlation coefficients (SCCs) are derived from the normalized ISI autocorrelation function, and estimate the average degree to which ISIs are correlated or anticorrelated with other ISIs in the sequence. SCC values were calculated as 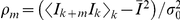, where *m* denotes the number of intervening intervals (lags), and ranged from 0 to 100 [33]. SCCs can range from −1 (perfect anticorrelation) to +1 (perfect correlation), while a value of 0 signifies no correlation. Spike generation is referred to as a renewal process if all SCCs in the spike sequence for m≥1 are 0, i.e., the ISIs are statistically independent.

Power spectral density (PSD), 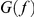, is a measure of the distribution of a signal's energy in the frequency domain. It is particularly useful for identifying periodicities in a signal, expressed by peaks at particular frequencies. For purposes of PSD calculations, each neuronal spike train was represented as a sequence of Dirac delta functions centered at spike times 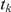 from which the mean firing rate 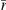 has been subtracted, 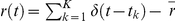. The PSD of a spike train, 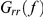, has units of (spikes/s)^2^/Hz or simply Hz. The delta functions were approximated by rectangular pulses of height 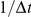, where 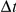 is the sampling interval, 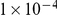
s. The PSD was then estimated using the Welch method (function *pwelch* in the MATLAB Statistical Toolbox) with a 2.048 s Hamming window.

#### Information measures

We used two approaches to assess information encoding in neural responses to external stimuli. The first was a conventional linear reconstruction technique that estimates the lower bound of the mutual information rate, 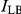 [23], [34]. In this approach, a Gaussian stimulus, *s(t)*, is applied to a neuron, and an estimate of the stimulus, 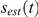, is obtained from the neural response by convolving the output spike train with an optimal linear filter that minimizes the SD of the noise in the reconstruction, calculated as 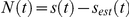. The characteristics of the optimal filter are specified by its transfer function, 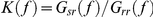, where 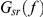 is the cross-spectral density of the stimulus and response, and 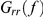 is the PSD of the stimulated spike train (response). The lower bound of the mutual information rate is estimated from the SR coherence function [35] as: 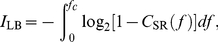
(1)where *f_c_* is the stimulus cutoff frequency. SR coherence is a normalized measure of stimulus-response cross-correlation at different frequencies, defined as: 
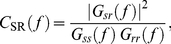
(2)where 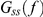 is the PSD of the stimulus. Calculations of SR coherence were done using the MATLAB function *mscohere*, with windowing as for PSDs.

The quality of the reconstruction is quantified by the coding fraction, 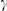, defined [30] as: 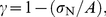
(3)where *A* is the SD of the stimulus, and 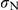 is the SD of the reconstruction noise, which can be calculated from the SR coherence [30] as: 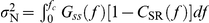. The coding fraction ranges from 0 (encoding on a chance level) to 1 (perfect encoding).

The second information metric we used was response-response (RR) coherence, which provides an estimate of the upper limit of the mutual information rate [26], [36], [37]. In this method, a neuron is stimulated by a sequence of identical segments of a Gaussian noise stimulus. Each stimulus segment results in a response 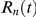. The average coherence between responses is: 
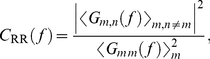
(4)where 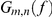 is the cross-spectral density of *m*-th and *n*-th responses, 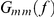 is the PSD of *m*-th response, and angled brackets indicate averaging over the ensemble of responses. The square root of RR coherence serves as the upper bound of SR coherence, so that the following inequality holds: 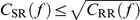.

### Numerical jitter analysis

A jittered response was obtained by adding independent zero-mean Gaussian random time offsets 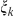 to each spike time. After computing the SR coherence for the jittered spike train, 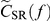, we obtained the lower bound of the mutual information rate, 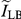, and the coding fraction, 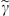, and compared them to 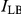 and 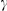
of the original spike train. This was repeated for various values of jitter SD, 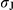, its magnitude. The tilde symbol denotes measures calculated from the jittered spike train.

### Theory for jittered spike trains

The derivations of equations used in this section to express the exact relationships between statistical metrics of original and jittered spike trains are given in Appendix S1. In the analysis that follows, we assumed that both the stimulus and response are stationary stochastic processes, and we used zero-mean Gaussian-distributed jitter with values that followed the real-valued characteristic function: 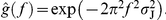. (5)

The coefficient of variation of a jittered spike train is: 
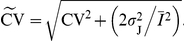
The SD of the jitter can conveniently be expressed in units of the SD of the original ISIs, 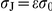, where 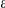 is a dimensionless scaling parameter. Using this substitution, the CV of a jittered spike train becomes: 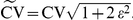. (6)

The serial correlation coefficients of the jittered ISIs, expressed in terms of the SCCs of the original ISI sequence, are given by: 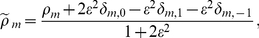
(7)where 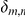 is the Kronecker delta function: 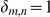 for 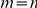, and 0 otherwise. The power spectral density function of a jittered spike train is: 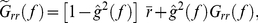
(8)where 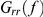 is the PSD of the original spike train. For normally distributed jitter with the characteristic function given by Eq.(5), the PSD of a jittered spike train will be: 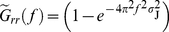
(9)


The cross-spectral density of a stimulus and a jittered spike train is: 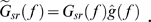
(10)


The SR coherence of a jittered spike train is: 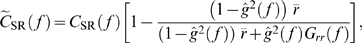
(11)and the RR coherence of a jittered spike train is: 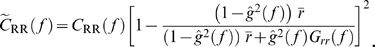
(12)


Equations 6–12 are exact and allow an investigator to calculate the metrics of a jittered spike train while bypassing the actual numerical procedure of jittering. For example, Eq. 11 with Eq. 1 allow analytical computation of the lower bound estimate of the mutual information rate for jittered spike trains, based solely on measures of coherence, firing rate and PSD of the original spike train.

### Model neurons with gamma-distributed ISIs

In a class of models, we simulated a spike train as a renewal process, i.e., where all ISI durations are independent, with an ISI probability density function (PDF) given by the gamma distribution, 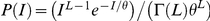, where the parameter *L*, called the *order* of the gamma distribution, sets its shape, and *θ* is a scaling parameter. The CV, mean ISI, and the ISI variance are, respectively, 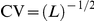, 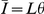, and 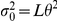. The PSD of the gamma spike train can be calculated exactly as: 
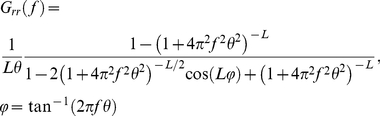
(13)


For large values of *L* (i.e., small values of CV), the PSD shows a sharp peak at a frequency corresponding to the mean firing rate, and smaller peaks at higher harmonics. Thus, for large *L*, the gamma neuron serves as a simple model of a regularly firing neuron. The special case where 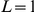 corresponds to a Poisson (random) spike train with a uniform power spectrum 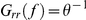.

A stimulus, 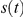, was introduced as Gaussian modulation of the firing rate, 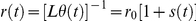, where 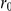 is the spontaneous firing rate, set to 50 or 100 spk/s. The Gaussian stimulus was band-limited to below a cutoff frequency 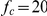
Hz, and had a flat PSD, 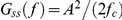, where *A* is the stimulus SD. Using this stimulus for a total duration of 600 s, we numerically generated sequences of ISIs for a rate-modulated gamma distribution.

### Phase model for neuronal oscillator

The dynamics of a periodically firing neuron responding to a stimulus, 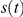, can be represented in terms of its phase [28], [29], [38], [39] as: 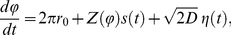
(14)where 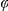 is the phase, 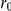 is the spontaneous firing rate, 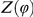 is a 2 π-periodic function known as the phase resetting curve (PRC), 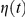
is a white Gaussian noise, and *D* is the noise intensity. Such a model generates a spike when the phase variable crosses the value of 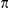 with a positive slope. We used the so-called type-I PRC where 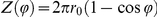, with spontaneous rate 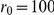
spk/s. The noise intensity was 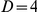, so that in the absence of any stimulus the model generated a spike train with CV = 0.045. The stimulus *s*(*t*) was identical to the one used for the gamma neuron, i.e. Gaussian, band-limited to below 20 Hz, with SD = *A*. The equation for *ϕ* was solved numerically using the explicit Euler method with a time step of 0.01 ms.

## Results

We developed an analytical framework (Methods, Appendix S1) to investigate the effects of jitter on spike train metrics commonly used to analyze sensory encoding and higher order statistics. We applied this analytical framework to data from model neurons and also experimental data from two types of sensory afferent neurons, to test the efficacy of jitter in distinguishing rate coding from temporal coding.

### Examples of jitter influences on stimulus encoding

**Figure 1:**
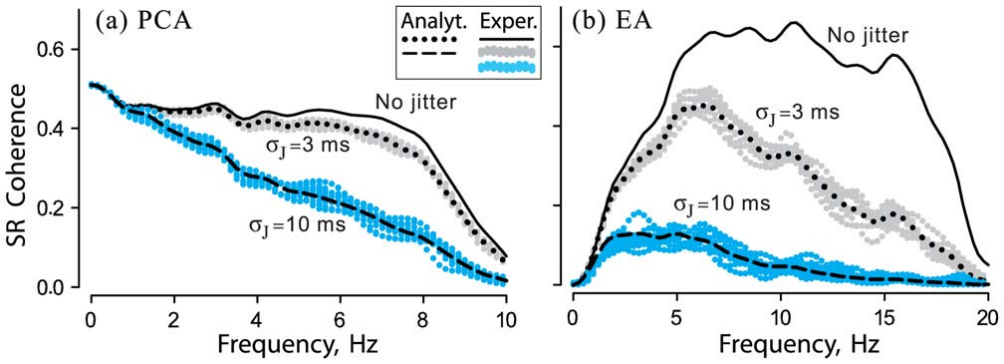
Effects of external spike time jitter on stimulus-response (SR) coherence. Data are shown for a turtle posterior canal vestibular afferent (PCA) (**a**), and for a paddlefish electroreceptor afferent (EA) (**b**). Stimuli were weak Gaussian noise, band-limited to below 10 Hz (a) or 20 Hz (b). 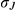: SD of jitter, 3 or 10 ms. *Exper.*: experimental results, for original spike trains (*black solid lines*), and also direct application of jitter to spike times (*cyan or gray shading*) repeated 10 times using different seeds of a random number generator. *Analyt.*: the dotted or dashed black lines show analytical results calculated from Eq.11, for 3 or 10 ms jitter SD.

**Figure 2:**
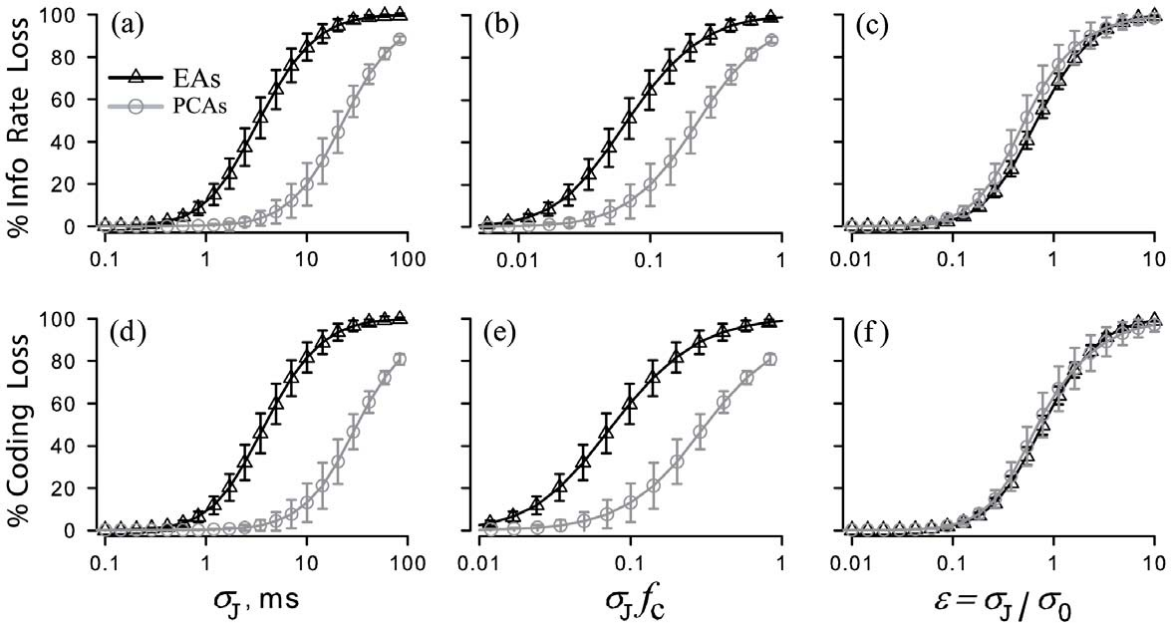
Reduction of mutual information and coding fraction due to spike time jitter. Percentage loss of the mutual information rate (**a–c**) and coding fraction (**d–f**), due to jitter, are shown over a wide range of jitter SD, expressed in 3 different ways (left, center, and right columns; see text), for n = 10 different EAs and n = 10 different PCAs. Eqs. 2, 3, and 11 were applied to spike time data from each afferent, to calculate analytically the effects of different-size jitter. This approach gave results indistinguishable from direct jittering of spike times. *Symbols and error bars*: mean ± SD, *n* = 10, for analytical results at evenly spaced (on logarithmic scales) values of jitter SD. *Continuous black or grey lines*: mean (*n* = 10) analytical results at intermediate values of jitter SD. *PCA sample*: spontaneous firing 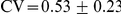, mean firing rate 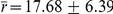 spikes/s, and SD of ISIs 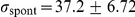 ms. *EA sample*: spontaneous firing 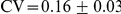, 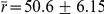 spikes/s, and 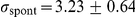 ms. *Stimuli* were Gaussian noise, band-limited to below 10 Hz for PCAs, or 20 Hz for EAs.

To set the stage, Fig. 1 illustrates the effects of jitter on experimental data from two representative examples of afferent neurons stimulated by relatively weak external Gaussian noise. The first example is a turtle posterior canal afferent (PCA) stimulated by mechanical indentation of the posterior semicircular duct. The stimulus had a SD of 6.7 µm and was band limited with an upper cutoff frequency 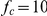 Hz (see Methods). The second example is a paddlefish ampullary electroreceptor afferent (EA) stimulated by a spatially uniform electric field with SD = 0.70 µV/cm and cutoff frequency 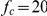 Hz. The PCA, with a background firing rate of 21.6 spk/s and CV = 0.21, had a higher intrinsic noise level than the EA which had a background firing rate of 49.0 spk/s and CV = 0.13. These differences are representative of the two afferent populations (see legend of Fig. 2).

SR coherence functions for these two afferents illustrate the effects of jitter in the frequency domain. For the PCA, jitter with SD≥10 ms resulted in a significant decrease of SR coherence, but smaller (e.g. 3 ms) jitter had minimal effects (Fig. 1a). In contrast, the EA showed a high sensitivity to jitter: SDs as small as 3–5 ms suppressed coherence dramatically (Fig. 1b). Note the excellent correspondence between the results obtained by numerically jittering the afferent spike times (shading) and the results obtained by analytic calculations using Eq. 11 (dotted and dashed lines).

Jitter analysis applied to samples of 10 PCAs and 10 EAs confirmed that, for a given jitter SD, PCAs were less sensitive to jitter than EAs. For the original responses, the lower bound of the mutual information rate (*I*_LB_) was 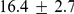 bit/s for the PCA sample, and 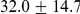 bit/s for the EA sample, while the coding fraction (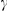) was 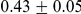 for the PCAs, and 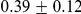 for the EAs (mean ± SD). Small jitter (SD = 5 ms) had little effect on PCAs, producing a 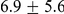
% reduction of *I*_LB_ values, and a 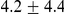
% reduction of 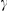 values (*circles*, Fig. 2a,d). However, for the EAs (*triangles*, Fig. 2a,d), jitter of identical SD had a much larger effect, reducing *I*_LB_ by 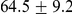
% and 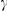 by 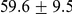
%. Because the SD of this jitter was much shorter than the time scale of the stimulus (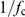 = 100 ms for PCAs, 50 ms for EAs), this result could indicate that EAs employ a temporal code to adequately represent the stimulus. In contrast, precise spike timing seems to be less important for PCAs [31].

The percent reduction for both the lower bound of the mutual information rate and the coding fraction increased sigmoidally with increasing jitter SD (Fig. 2). When jitter SD is expressed in units of time, most effects are seen to occur over a 10-fold size range, although the boundaries of the effective range differs between the two types of afferents (Fig. 2a,d). To determine if jitter effects were simply related to the cutoff frequency of the stimulus, the reductions of *I*_LB_ and *γ* were also plotted (Fig. 2b, e) after normalizing the jitter SD to the time scale of the stimulus, 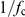. The fact that the curves for the PCA and EA (in Fig. 2b, e) do not superimpose indicates that jitter effects are not simply related to the proximity of the jitter SD to 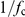. To assess any relationship between jitter size and the intrinsic variability of a neuron, we also normalized jitter SD to the SD of the afferent ISI distributions. This type of normalization resulted in nearly identical information and coding loss curves for the two afferents (Fig. 2c,f). This indicates that the intrinsic variability of a neuron is the principal determinant of its sensitivity to external jitter. For both afferent samples, decreases in the mutual information rate and the coding fraction exceeded 50% at the point where the jitter SD equaled the SD of the afferent ISI distributions. Since both *I*_LB_ and 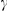 showed a similar strong dependence on jitter SD, we consider only *I*_LB_ in the following sections.

### Influences of jitter on serial correlations among ISIs

Adding jitter to a spike train indeed increases the variability of its ISI distribution, which is reflected in the increase of CV with jitter SD according to Eq. 6. For renewal processes of similar mean firing rate, the effect of jitter is stronger for less variable spike trains, i.e., those with smaller CV values (more uniform ISIs).

 Jitter also alters the serial correlation coefficients among ISIs in two different ways. First, the striking result of Eq. 7 is that *jitter introduces negative serial correlations* into ISI sequences that were originally generated by a renewal process. For example, with jitter, the SCC of adjacent ISIs, i.e., where lag, *m* = 1, becomes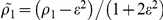, where 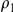 is the SCC of the adjacent ISIs in original spike train, and 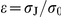, where 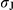 is the jitter SD and 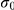 is the SD of the original ISI distribution. If the original spike train is a renewal process, where 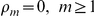, then the first SCC of the jittered spike train, 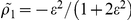, becomes negative and approaches −0.5 for large values of *ε* 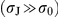. Thus, jitter converts a renewal process to a non-renewal process in which adjacent ISIs are negatively correlated, i.e., short ISIs will tend to be followed by long ISIs and *vice versa*. This introduction of anti-correlated sequential ISIs can be understood qualitatively as a consequence of the jitter values that are added to spike times being drawn from a distribution with a mean. For any pair of spikes, a large absolute value of jitter added to the first spike time is more likely to be followed by a jitter value closer to the mean being added to the second spike time, due to the phenomenon of regression (reversion) to the mean. Thus, the *interval* between the spike pair is modified by a pair of numbers that themselves tend to be anti-correlated.

Second, a quite different effect of jitter is observed for spike trains that are originally generated by a non-renewal process. In this case, the non-zero SCC values of the original spike train are *suppressed* by jitter: 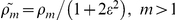. Since 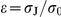, this scaling parameter in the denominator means that larger jitter brings the SCC values 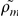 closer to zero. This effect is also stronger for less-variable spike trains.

**Figure 3:**
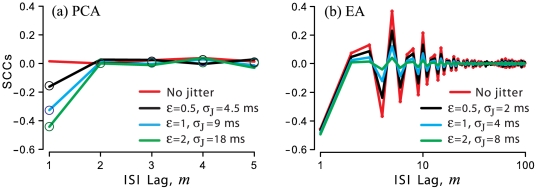
Influence of spike time jitter on serial correlation coefficients (SCCs). SCCs of spontaneous firing of one PCA (**a**) and one EA (**b**), are shown for the listed values of jitter SD, 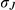, also expressed as a multiple, *ε*, of the SD of the original ISI distribution. *Open circles,* (*a*): theoretical predictions of Eq. 7. *Abscissa*, (*b*): note the logarithmic scale of the ISI lag number, *m*.

Fig. 3 illustrates these effects of jitter on SCCs of spontaneous spike trains of a typical turtle PCA and a typical paddlefish EA. These data are complementary in that turtle PCAs show renewal statistics [40] whereas paddlefish EAs exhibit extended-range serial correlations due to interaction of two distinct types of embedded oscillators [21], [41]. Jitter introduced a negative SCC at the first lag (*m* = 1) for the renewal afferent (PCA, Fig. 3a), and the value of this SCC became more negative for larger jitter SD, as expected. By contrast, for the non-renewal afferent (EA, Fig. 3b), larger jitter resulted in greater suppression of SCCs for *m*>1, as expected.

### Influences of jitter on spike train power spectra

**Figure 4:**
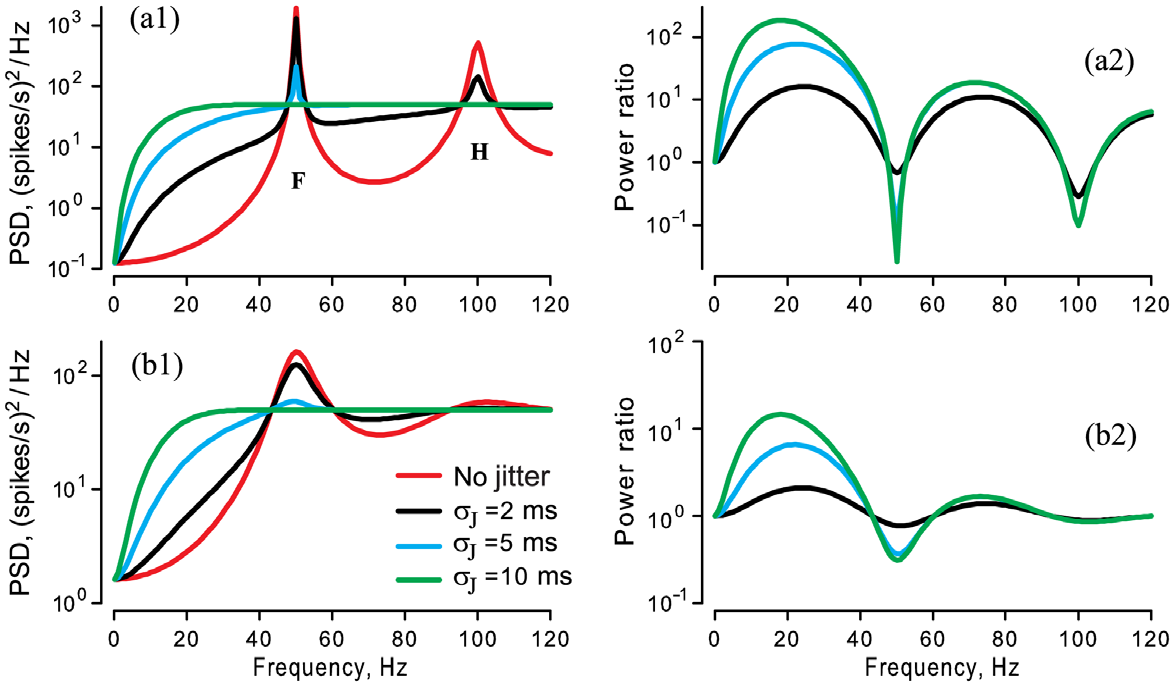
Effect of spike time jitter on the power spectral density (PSD) of spontaneous neural discharge of two different model gamma neurons calculated from Eq.13. For both, the mean firing rate was set to 50 spike/s. **a1, a2**. The gamma distribution's order was 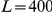 resulting in CV = 0.05 and 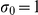
ms for the SD of the original ISIs. **b1, b2**. 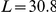 resulting in CV = 0.18 and 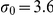
ms. (**a1, b1**) *Red lines:* PSD of original spike trains given by Eq.13. *Black, green, blue lines*: PSDs of jittered spike trains given by Eq.9, for the listed values of jitter SD, 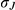. In (a1), the fundamental (F) peak and its harmonic (H) are labeled. (**a2, b2**) Corresponding power ratios, 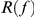, of PSDs for jittered ÷ original spike trains.

**Figure 5:**
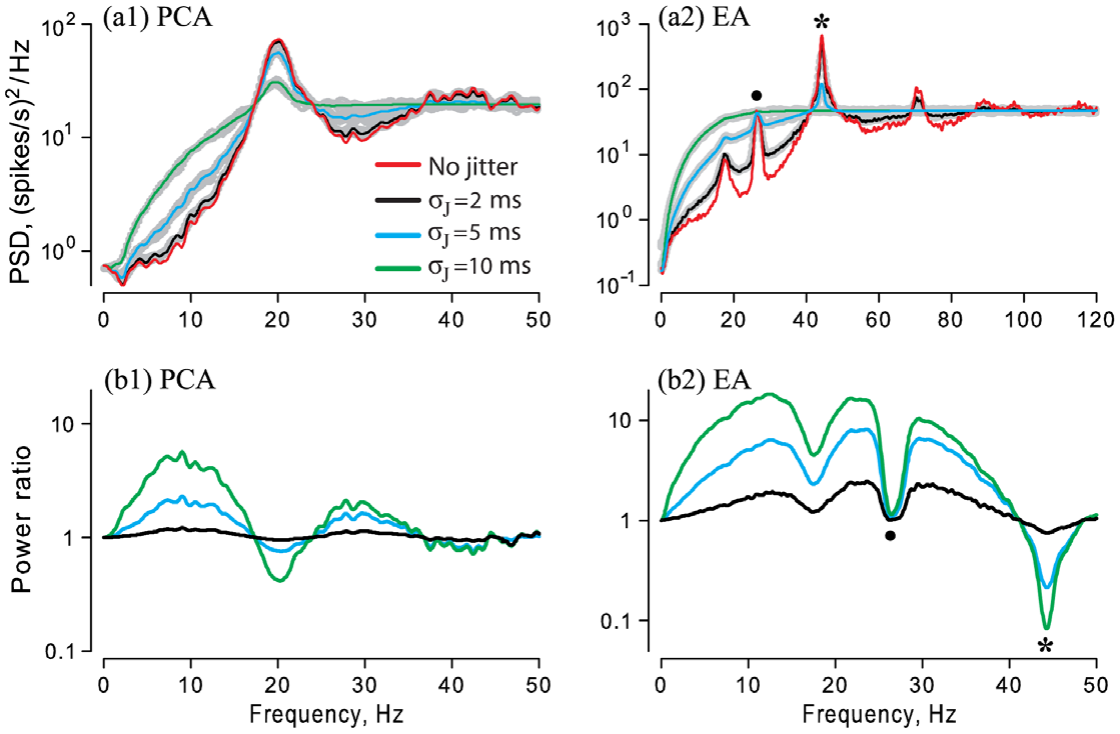
Effect of spike time jitter on PSD of spontaneous discharge of one PCA (a1) and one EA (a2). *Grey shading*: direct application of jitter to spike times, with listed values of jitter SD, 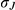, using 10 different seeds of a random number generator. *Red lines:* PSD of original spike trains. *Black, green, blue lines*: analytical results from Eq. 9, for the listed values of jitter SD, 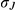, superimposed on corresponding grey shading for each. (**b1, b2**) Corresponding power ratios 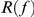. Same afferents as for Fig. 3. The PCA and EA had matching CV = 0.18, but different mean firing rates, 19.6 spike/s for PCA and 44.3 spike/s for EA, yielding SD values of spontaneous ISI distributions of 9 ms and 4 ms, respectively. *Asterisk, dot*: see text.

 The power spectrum of a regularly firing neuron has a main peak at a fundamental frequency corresponding to the mean firing rate, and broader peaks at harmonics of the fundamental, as seen in Fig. 4 (labels **F** and **H**) for a model neuron with gamma-distributed ISIs, and in Fig. 5 for spontaneous PCA and EA spike trains. As the CV decreases, the peak at the fundamental frequency becomes narrower and higher (Fig. 4, red line in a1 vs. b1, for CVs of 0.05 vs. 0.18), other factors being equal. The discharges of the gamma neuron model (Fig. 4) and the PCA (Fig. 5a1) are both renewal processes, i.e. lacking any serial ISI correlations. In contrast, the EA's spontaneous discharge is non-renewal due to the interaction of multiple internal oscillators [21], and so the PSD of the EA (Fig. 5a2) shows several additional peaks besides at the mean firing rate (*asterisk* at 44.3 Hz), including one due to the epithelial oscillations (*dot* at 26 Hz), and peaks at second-order combinations of these fundamentals (44.3±26 Hz) [21].

As Figs. 4 and 5 show, the PSDs of original and jittered spike trains converge for extremely low frequencies (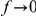). They also converge for very high frequencies (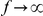) because the PSD, 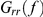, approaches 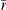 for original as well as jittered spike trains (Eq.11). At intermediate frequencies, jitter increases power, except around the mean firing rate and its higher harmonics, where power is suppressed by the jitter. In other words, external jitter both enhances intrinsic variability and suppresses intrinsic periodicity of discharges.

A non-obvious outcome of our analysis is that, for a regularly firing neuron, even small millisecond-level jitter results in a dramatic increase in PSD power at frequencies below the mean firing rate, if 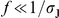. This increase of power is important for our purposes because the major fraction of stimulus power often lies in this low frequency range. To further quantify this, we considered the power ratio of the PSDs of the jittered and original spike trains, 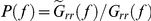, where 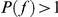 indicates a power gain produced by jitter. The power enhancement at low frequencies is more dramatic for afferents with more regular discharge, as seen in comparing 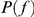 for the gamma neuron with CV = 0.05 (Fig. 4a2) or CV = 0.18 (Fig. 4b2).

Small (2–5 ms) jitter caused analogous changes in the PSD of an EA (Fig. 5a2), including a more than 10-fold power gain at low frequencies (Fig. 5b2). Similar but less pronounced effects were observed for the PCA for small jitter (2–5 ms) (Fig. 5a1 and 5b1). This EA and PCA had matching CV = 0.18. Larger jitter (10 ms) completely abolished this EA's PSD peaks.

 Our analytical results provide a clear explanation of this power gain at low frequency, as indicated by the excellent correspondence between numerical results from direct jittering procedures and analytical results from Eq. 9 (gray shading vs. superimposed lines in Figs. 5a1 and 5a2). For low frequencies and small jitter, 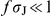, the Taylor expansion of Eq. 9 with terms up to 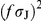 gives a PSD for the jittered spike train of: 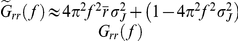. The first term in this expansion indicates an increase in power at low frequencies for the jittered spike train, proportional to the square of the frequency. This is visible in Figs. 4a1 and 4b1 in the steep initial slopes of jittered curves, just above zero frequency. A similar Taylor expansion of the power ratio gives:
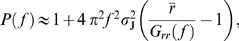
(15)


which shows that at low frequencies the power ratio also scales as the square of the frequency.

The magnitude of the power gain is determined jointly by the jitter SD and by the variability of the original spike train, i.e. the term inside brackets in Eq.15. At zero frequency, the PSD of a spike train is determined by the CV, the mean firing rate, and the sum of the SCCs: 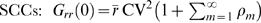 [33]. For a regular neuron with a small CV, 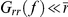 at low frequencies (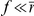). Thus, from Eq. 15, the increased power gain due to jitter is more dramatic both for more-regular neurons with smaller values of CV, and for neurons with higher values of the mean firing rate.

If present, negative SCCs further suppress low-frequency variability, resulting in reduced spectral power at low frequencies, compared to a renewal process of equal CV and mean firing rate [42], [43]. Consequently the presence of negative SCCs will cause the effects of jitter to be stronger even if the CV is relatively large, e.g. for the EA in Figs. 3 and 5.

### Comparison of jitter effects on SR coherence and transfer functions

Besides reshaping the PSD of spontaneous spike trains (Figs. 4 and 5), the addition of jitter also decreases a response metric to external stimulation, the SR coherence. This is easy to see from the definition of the SR coherence function, Eq. 2, where the spike train PSD is in the denominator, such that the jitter-induced increase of power within the frequency band containing the stimulus leads to a decrease of SR coherence.

For low frequencies and Gaussian jitter, 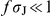, the SR coherence function of a jittered spike train can be expanded to a Taylor series, 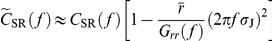



This shows that the reduction of SR coherence by jitter is lessened by neuron-specific variability, which is proportional to 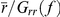 as discussed *above*. For a given value of jitter SD, the reduction of SR coherence and the mutual information rate will be greater for a regular spike train, because the magnitude of spike train power 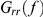 will be smaller (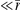) within the low frequency band of a stimulus (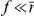). This explains the difference between the magnitude of the jitter effects at low frequencies observed for the PCA and EA in Fig. 1.

On the other hand, another metric of responses to stimulation, the transfer function 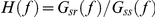, is less affected by jitter. 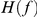 is a ratio expressing the response magnitude of a linear system relative to the power of a stimulus, at different frequencies. It is not normalized to the spike train PSD, and so is less affected by jitter. The expansion of 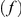 to a Taylor series for 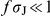, 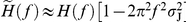, shows that the effect of jitter on the transfer function does not depend on the variability of the original spike train at all, and is small for 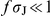. This has been observed experimentally [24].

#### Considerations from Linear Response Theory

 Further insight into the effects of jitter came from using linear response theory [44] to approximate the SR coherence function [28], [43], [45]. In this approach, for weak stimuli, the PSD of a stimulated spike train is approximated as the sum of the PSD of the spontaneous discharge 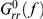 and the PSD of the stimulus weighted with the square of the transfer function 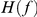: 
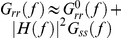
. Consequently the SR coherence becomes: 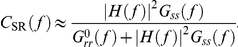
At low frequencies and small jitter magnitudes, 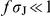, only the term for the PSD of the spontaneous discharge is strongly affected by the jitter. Thus, for weak stimuli, the suppression of SR coherence at low frequencies by jitter is explained entirely by jitter's effect on the PSD of spontaneous discharge, without any reference to the stimulus.

#### Model neuron with gamma-distributed ISIs

**Figure 6:**
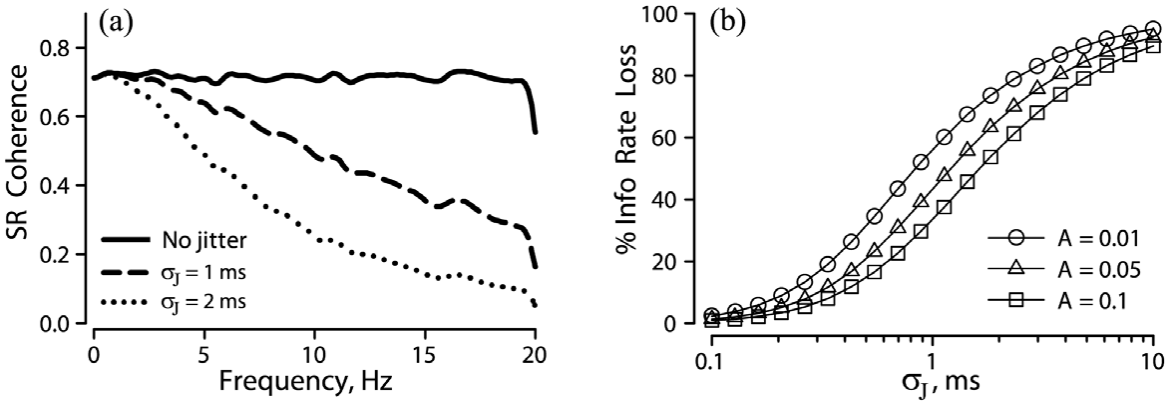
Effects of small-magnitude spike time jitter on stimulus–response (SR) coherence for a model gamma neuron. (**a**) Reductions observed for two values of jitter. Background firing rate, *r_0_* , was 100 spikes/s, modulated by a Gaussian noise stimulus that was band-limited to below 20 Hz. CV = 0.044 for background firing, corresponding to a parameter value of *L* = 520 for this gamma distribution (see Methods). (**b**) Percentage loss of mutual information rate as a function of jitter SD, 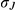, for the 3 listed values of stimulus SD = *A*.

To demonstrate explicitly that information carried by *rate-modulation* of a spike train is sensitive to small external jitter, we constructed spike trains from ISI sequences generated from a gamma distribution, with the spike rate modulated by a slow Gaussian stimulus (Methods). To mimic the situation of the mammalian vestibular afferents studied in Sadeghi et al. [24], we constructed spike trains with a spontaneous rate of 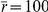 spk/s and 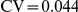. The firing rate was modulated by Gaussian noise with a cutoff frequency of 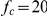 Hz (Methods). Fig. 6a shows that jitter with SD as small as 1 ms significantly suppressed the SR coherence and, consequently, the mutual information rate. This demonstrates clearly that reduced stimulus encoding resulting from the addition of small external spike time jitter can be observed in the absence of any temporal code.

**Figure 7:**
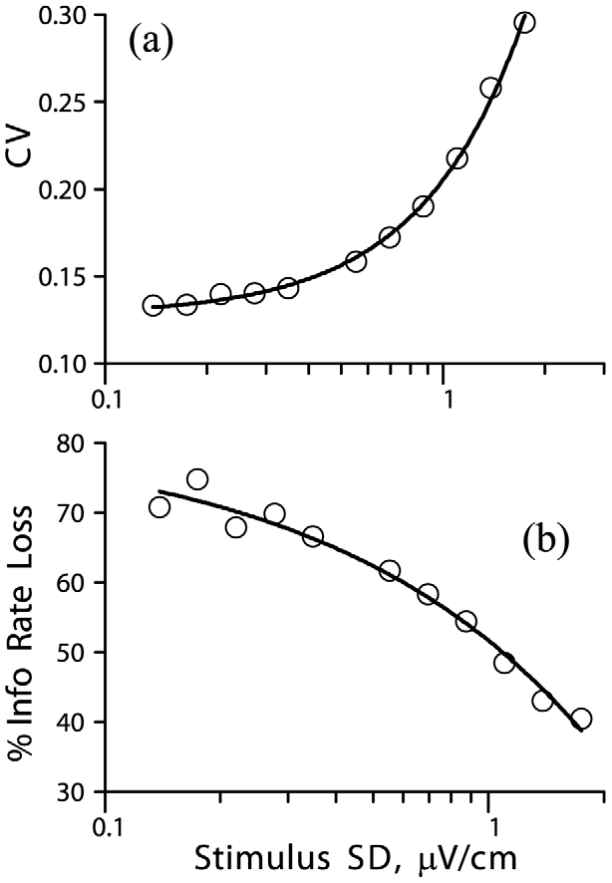
Effect of stimulus SD on coefficient of variation (CV) and mutual information. (**a**) CV of a spike train, vs. SD of a noise stimulus, without jitter, for one EA. (**b**) Percentage loss of mutual information rate due to 3 ms jitter; same data as in panel (a). The *solid line* in each panel shows a least squares fit with quadratic polynomials. Same EA as in Fig. 1.

How does stimulus magnitude affect the sensitivity of a spike train to external jitter? For a weak Gaussian noise stimulus alone, the low-frequency power in the spike train will increase with stimulus amplitude and the CV of the spike train will increase quadratically with stimulus SD (Fig. 7a). The effect of jitter alone can be represented as an increase in ISI variability (CV). According to our analysis (*above*, Fig. 2c,f), the sensitivity of a spike train to external jitter decreases as the variability of the original spike train increases. Thus, our analysis predicts that the effects of jitter of a given magnitude will become smaller as stimulus SD increases. This prediction was borne out for a model gamma neuron (Fig. 6b), and for an EA (Fig. 7b): for a fixed value of jitter SD, the percentage of information loss due to the jitter decreased for larger values of stimulus SD.

### Effect of jitter on nonlinear responses

**Figure 8:**
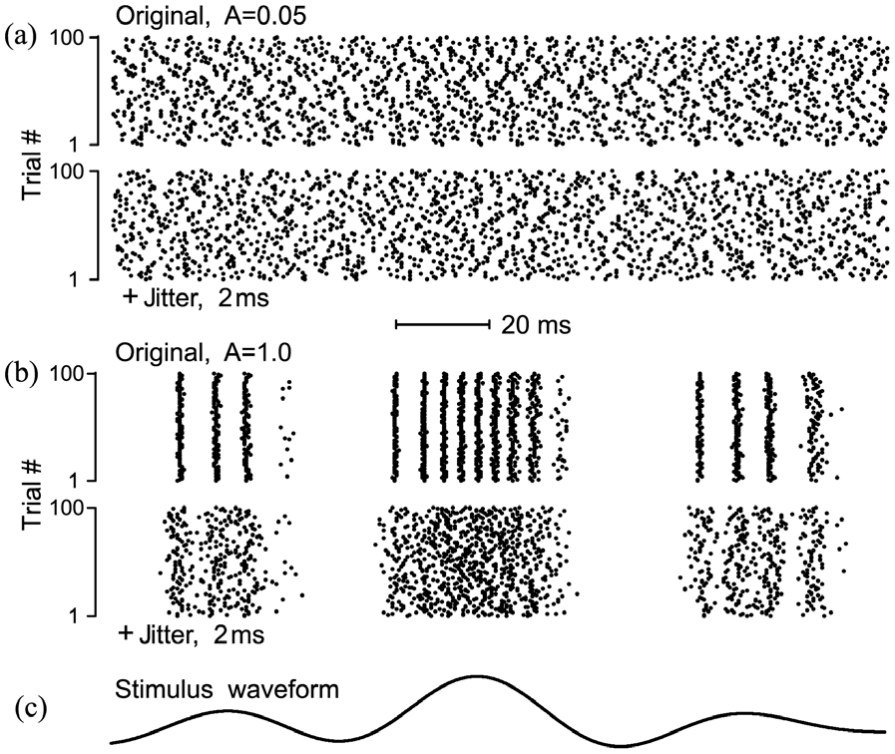
Effects of spike time jitter on linear (a) and nonlinear (b) responses from a phase neuron model (Eq.14). The parameters of the model were chosen to match the model gamma neuron of Fig. 6: 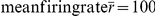
spike/s, CV = 0.045, and 20 Hz cutoff frequency of a Gaussian noise stimulus. The *upper* block of panels (a) and (b) shows 100 raster plots of spike times (*dots*) during 100 presentations of an identical 180 ms stimulus segment (**c**), taken from a 600 s stimulus waveform that was repeated. The raster plots in the *lower* block of each panel show the effect of adding 2 ms jitter to spike times, for the same stimulus SD, *A*, which was ‘weak’ (*A* = 0.05) for the responses in (a), and ‘strong’ (*A* = 1.0) for the responses in (b).

Finally, we wish to determine if the effects of jitter on linear responses can be dissociated from the effects of jitter on non-linear responses, where precise spike timing carries information in addition to that carried by firing rate. As the amplitude of a stimulus grows, the response of a neuron becomes progressively nonlinear, and a linear encoding model is no longer optimal [36]. One approach to revealing such nonlinear responses in neurons is to repeatedly present an identical segment of a noise waveform, so-called “frozen noise”. If the stimulus is strong enough, individual spikes become time locked to particular stimulus features resulting in stereotypical neural responses to repeated stimulus presentations [46]–[49]. We studied the effects of jitter on stimulus-induced synchronization using a phase neuron model (Eq. 14). Fig. 8 shows raster plots of this phase model's spike times in response to repeated presentations of 600 s segments of weak or strong “frozen noise” stimuli, as well as the effects of adding 2 ms jitter. For the weak stimulus alone (8a, *upper block*), spike times varied across stimulus trials because of intrinsic noise in the system, and the jitter has no apparent effect (8a, *lower block*). However, for the stronger stimulus (8b, *upper block*), the phase model's firing was tightly locked to the stimulus so that spike times were synchronized into well-defined temporal patterns, reproduced reliably across the ensemble of stimulus trials. For this stronger stimulus, the 2 ms jitter clearly degraded the spike time synchronization (8b, *lower block*).

**Figure 9:**
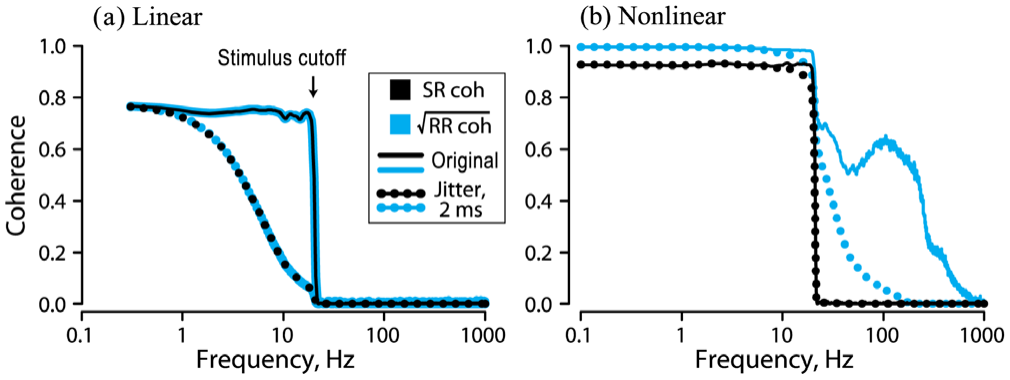
Stimulus-response (SR) and response-response (RR) coherence for the linear and nonlinear regimes shown in Fig. 8. *Solid* lines: original data. *Dotted* lines: with small spike time jitter (SD = 2 ms) added. SR coherence is shown by *black* lines and dots. RR coherence is shown by *blue* lines and dots.

We quantified the effects of jitter on this cross-trial synchronization by comparing SR and RR coherences for the original and jittered spike trains (Eqs. 2, 4, 11, 12). For a weak stimulus, SR and RR coherence were essentially identical and can hardly be distinguished in Fig. 9a (black vs. blue *solid* lines), indicating that a linear stimulus encoding model is appropriate. Small (2 ms) spike time jitter resulted in identical and significant reduction of both coherence functions (Fig. 9a, black vs. blue *dotted* lines), that was nearly complete by the stimulus cutoff at 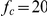 Hz.

For a stronger but otherwise identical noise stimulus (Fig. 9b), the response was clearly nonlinear, indicated by RR coherence (*blue solid line*) being larger than SR coherence (*black solid line*) across the whole frequency range. The large values of RR coherence at high frequencies up to 500 Hz, well beyond the stimulus cutoff at 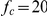 Hz, clearly reflected the stimulus-induced spike synchronization and the resulting small trial-to-trial spike time variability seen in Fig. 8b (*upper block*). For this nonlinear response, small jitter (SD = 2 ms) affected SR and RR coherence in different ways. There was insignificant suppression of SR coherence (Fig. 9b, *black dotted line*) by such small jitter across the entire frequency range, due to the large intra-trial variability of ISIs, that is, their large CV imposed by the stimulus. On the other hand, RR coherence (*blue dotted line*) was strongly suppressed, but only in the high-frequency band, 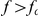, indicating that small jitter values contaminated only the trial-to-trial spike synchronization.

## Discussion

Spike generation is an inherently noisy process, due to various internal sources of noise in neurons [46], [50]–[52]. Consequently the temporal precision of neuronal responses is always somewhat degraded by intrinsic spike time jitter. Indeed, estimating and removing this inherent jitter from neural responses has been shown to significantly improve stimulus reconstructions and estimates of neural transfer functions computed from spiking responses [9]. A reverse approach is often used to assess the degree to which the application of *artificial* jitter (noise) to the timing of spikes in a spike train degrades stimulus encoding [6], [20], [24]–[26]. We have developed an analytical framework that allows quantitative assessment of the effects of artificial spike timing jitter on both the spontaneous and stimulus-driven discharges of sensory neurons. This framework allows an efficient and analytical assessment of the effects of jitter on various spike train metrics, without requiring the actual numerical jittering procedure. In particular, our theory applies to information metrics estimated using the linear stimulus reconstruction technique, as in numerous neuroscience studies.

We have applied this analytical framework to experimental data from turtle vestibular and paddlefish electroreceptor afferents, as well as to model neurons. Our main results regarding the effects of added spike time noise on sensory encoding are: **1.** Jitter increases the variability of spontaneous discharges, as measured by the CV. However, jitter also drastically reshapes the correlation metrics of a spike train, e.g. serial correlation coefficients, and the power spectrum. **2.** The *relative* size of jitter is a critical parameter, as jitter reduces stimulus-response coherence in proportion to the ratio of the jitter SD to the intrinsic variability of the neuron's discharge. **3.** Jitter reduces both the mutual information rate and the coding fraction of neuronal responses, even in cases where information is linearly encoded. For example, a gamma model neuron with the rate modulated by the stimulus (that is, with a strict rate-coding scheme explicitly imposed) is sensitive to jitter in the same manner as sensory afferents. **4.** For non-linear responses, where spikes are synchronized to stimulus events, small amounts of jitter that have minimal effect on SR coherence can indeed significantly reduce the cross-trial (RR) coherence of repeated responses, and non-linear encoding. Based on these results, we conclude that the degradation of sensory encoding resulting from added spike time noise, as estimated with the *linear* reconstruction technique, does not provide unequivocal evidence for a temporal code.

### Distortions from jitter

We showed that jitter alters the correlation structure of spike sequences. Jitter introduces negative serial ISI correlations into renewal processes, due to the zero-mean nature of the jitter size distribution. On the other hand, jitter degrades serial ISI correlations that are already present. Such distortions by jitter have not been described previously, but since the distinction between renewal and nonrenewal processes is considered fundamental by computational neuroscientists (reviewed in [53], [54]), these distortions may complicate the interpretation of jitter effects. Although negative serial correlations have been shown to reduce background noise in neural discharges [42], [43], [55]–[58], which can enhance linear encoding, the full impact of ISI correlations on CNS processing of sensory information and animal behavior is not known.

### What determines a neuron's susceptibility to small external jitter, and what is a meaningful criterion for “small”?

The rationale behind the use of jitter to demonstrate temporal encoding is that the jitter SD, 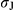, is assumed to be small relative to the duration of the neuron's encoding window. Thus, jitter alters the position of spikes within the window (a temporal code), but not the number (a rate code). In the absence of any direct information about the length of the encoding window, the jitter amplitude is typically chosen to be smaller than the characteristic time scale of the stimulus. For a Gaussian noise stimulus that is band-limited to a cutoff frequency 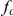, this criterion for small jitter is then: 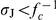, or 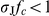. For example, jitter with an SD of 3 ms would be considered to be small for a stimulus with a cutoff frequency of 20 Hz, since 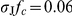.

In our results, jitter of this small magnitude resulted in a significant reduction of information measures for electroreceptor afferents, but not for vestibular afferents (Fig. 2a,d). This appears consistent with the degree of jitter-induced suppression of information metrics being determined by the *intrinsic* variability of a spontaneously active neuron, such that neurons with a more regular discharge appear more susceptible to small jitter.

The suppressive effect of jitter on stimulus encoding becomes obvious when the jitter SD is approximately equal to the standard deviation of the neuron's ISI distribution, 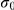, that is, when 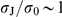 (Fig. 2c,f). This result establishes a completely different criterion for what constitutes small jitter. For the example of the electroreceptor afferents with 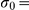
3–5 ms, jitter with 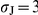 ms is not small by the new criterion, and indeed significantly reshapes the power spectrum and the SR coherence function of the afferent. On the other hand, for the vestibular afferents, a 3 ms jitter is small relative to its 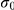 value (10–40 ms), and consequently it does not significantly affect the afferent's response.

Normalizing the magnitude of the jitter to the value of 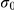, 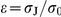, provides a universally applicable means of scaling the jitter magnitude for purposes of evaluating its effects on information theoretic metrics, as shown in Fig. 2c,f. This suggests that the effect of jitter is essentially independent of spontaneous discharge regularity *per se*, if normalized to it, and that the appropriate criterion for considering jitter to be small is unrelated to the time scale of the stimulus, 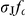. Thus, if scaled appropriately, artificial spike time noise has consistent effects on sensory encoding no matter whether a neuron's spontaneous firing is noisy or highly periodic, high or low frequency, renewal or non-renewal.

### Why does small-amplitude jitter affect the encoding of low-frequency stimuli?

We examined this question in detail. With the linear reconstruction technique, both the lower bound estimate of mutual information rate and the coding fraction are expressed in terms of SR coherence (Eqs. 1–3). Thus, the suppression of information encoding due to artificial jitter can be understood entirely in terms of the relationship between SR coherence and jitter SD. Our analysis shows that for low frequencies and small jitter amplitudes, i.e. when the product of the frequency and the jitter SD is small (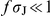), the suppression of SR coherence is due to an increase in the power spectral density of the jittered response, while the cross-spectrum is relatively unaffected. That is, artificial jitter significantly enhances power at frequencies lower than the mean firing rate of a regularly firing neuron, and much lower than the inverse of the jitter SD. For progressively weaker stimuli, this jitter-induced power gain is increasingly stimulus independent. Since the response PSD increases without any corresponding increase in the cross-spectrum, the SR coherence is reduced for the jittered responses, and consequently the lower bound estimate of the mutual information rate, and the coding fraction, are reduced also.

The same argument can be made for the optimal reconstruction filter, 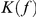, which is calculated as the ratio of the cross-spectrum to the response PSD (Methods), in the frequency domain. Here also, jittered responses exhibit enhanced power in the background noise, which reduces the magnitude of the reconstruction filter, and leads to a reduction of the mutual information rate and the coding fraction. Thus, the sensitivity of an encoding process to small-amplitude jitter, as estimated with the linear reconstruction technique, can be explained completely by jitter-induced transformations of the response spectral characteristics.

The example of Fig. 9a clearly shows that for a weak stimulus and a neural response in which all information is linearly encoded, dramatic suppression of the lower bound mutual information rate by 2 ms jitter can occur. Our example with a rate-modulated gamma neuron (Fig. 6a) further demonstrates that small jitter has essentially the same effect on responses that are explicitly rate encoded as it does on the afferent neurons used in this study. Thus, the sensitivity to small external jitter of stimulus encoding, as estimated by linear reconstruction, cannot *per se* be taken as evidence for a temporal encoding scheme.

### Jitter effect on nonlinear encoding

The effects of jitter on linear and non-linear encoding were quite distinct. For non-linear responses manifested as stimulus-induced synchronization of neuronal firing (as Fig. 8b), estimates of the lower bound of the mutual information rate were essentially unaffected by jitter (Fig. 9b). Instead, jitter dramatically reduced the cross-trial (RR) coherence between responses to a repeated stimulus, but only at frequencies above the stimulus band, corresponding to the time scale of the trial-to-trial spike synchronization induced by the stimulus. Thus, jitter does disrupt coding schemes based on spike timing, when present.

A full assessment of the effects of jitter requires that it also be applied to measures of non-linear encoding, such as direct estimates of the mutual information rate [7], [8], [59], or RR coherence and the upper bound of the mutual information rate associated with it [23], [26], [37].

The variability of neurons with sparse spontaneous activity, e.g. thalamic neurons [8] or whisker primary afferents [25], [27], as estimated from a single presentation of a noise stimulus, is mainly determined by the stimulus itself. Although our theory also applies to such neurons, the use of cross-trial (RR) variability to assess intrinsic variability may be more appropriate than the CV metric used here for afferents with robust spontaneous activity. Indeed, for neurons with sparse spontaneous activity, lower bound information estimates may significantly underestimate the true mutual information rate [10]. Measures of nonlinear encoding must be implemented instead [7], [8], [26], [37], [59].

## Supporting Information

Appendix S1 **Derivation of relationships between original and jittered spike trains.**
(DOC)
