# Supplementary material for: Identifying Temporal Codes in Spontaneously Active Sensory Neurons
Source: PLoS One. 2011 Nov 8;6(11):e27380. doi: 10.1371/journal.pone.0027380 (PMC3210806; doi:10.1371/journal.pone.0027380)
Supplement: Appendix S1 — Derivation of relationships between original and jittered spike trains. (DOC) [file pone.0027380.s001.doc]

**Appendix S1: Derivation of relationships between original and jittered spike trains**

Here are derived Eqs. 6 – 12, which relate various metrics of the original and jittered spike trains. The only assumption underlying this analysis is that the stimulus and the response are both stationary stochastic processes. A diacritical tilde symbol denotes quantities for jittered spike trains.

*Coefficient of variation (CV).* Jittered spike times are $\mathop{t_{k}}\limits^{\sim} =t_{k}+\xi_{k}$, where $\xi_{k}$ represents a zero-mean uncorrelated random time offset that is independent of $t_{k}$ and has an SD of $\sigma_{\text{J}}$. This can be formalized with cross- and autocorrelation functions, $\left\langle t_{k} \xi_{k}\right\rangle_{k}=0,$ $\left\langle\xi_{k+m} \xi_{k}\right\rangle_{k}=\sigma_{_{\text{J}}}^{2}\delta_{m,0}$. The jittered ISIs are $\mathop{I_{k}}\limits^{\sim} =\left(t_{k+1}+\xi_{k+1}\right)-\left(t_{k}+\xi_{k}\right)=I_{k}+\xi_{k+1}-\xi_{k}$. Since $\left\langle\xi_{k}\right\rangle=0$, the mean of the jittered ISI distribution is indeed the same as that of the original spike train. Also, since $\xi_{k+1}$ and $\xi_{k}$ are independent, the variance of the jittered ISIs equals the variance of the original ISIs plus $2\sigma_{_{\text{J}}}^{2}$. Thus, the CV of the jittered spike train is $\mathop{\text{CV}}\limits^{\text{\textasciitilde}} =\sqrt{\text{CV}^{2}+\left(2\sigma_{_{\text{J}}}^{2}/\bar{I}^{2}\right)}$, and using $\varepsilon=\sigma_{\text{J}}/\sigma_{0}$ we obtain Eq. 6 for the CV of the jittered spike train.

The *serial correlation coefficients (SCCs)* of the ISIs in a jittered spike train are: . Noting that $\left\langle\xi_{k+m}\xi_{k}\right\rangle_{k}=\sigma_{\text{J}}^{2}\delta_{m, 0} ,$ $\left\langle\xi_{k+m}\xi_{k+1}\right\rangle_{k}=\sigma_{\text{J}}^{2}\delta_{m, 1} ,$ $\left\langle\xi_{k+m+1}\xi_{k}\right\rangle_{k}=\sigma_{\text{J}}^{2}\delta_{m,-1} ,$ and , we obtain the SCCs of the jittered ISIs in units of the SCCs of the original ISIs: $\rho^{\sim} _{m}=\left(\rho_{m}+2\varepsilon^{2}\delta_{m, 0}-\varepsilon^{2}\delta_{m, 1}-\varepsilon^{2}\delta_{m,-1}\right)/\left(1+2\varepsilon^{2}\right)$, Eq. 7.

*Power Spectral Density (PSD).* For calculation of the PSD of a jittered spike train, we need to introduce *n*-th order interspike intervals, $I_{n,k}=t_{k+n-1}-t_{k-1}=\sum_{j=0}^{n-1} I_{j+k}$ [1]. Each *n*-th order ISI is characterized by its probability density function (PDF), $F_{n}\left(I\right)$. For $n=1$ we obtain $F_{n}\left(I\right)=F\left(I\right)$, the PDF of the conventional ISIs. The PSD of a stationary spike train, $G_{rr}\left(f\right)$, is expressed through the characteristic functions, i.e. Fourier transforms, of the PDFs of *n*-th order ISIs, $\widehat{F}_{n}\left(f\right)$, as in [2]:

$G_{rr}\left(f\right)=\bar{r}\left\{1+\sum_{n=1}^{\infty} \left[\widehat{F}_{n}\left(f\right)+\widehat{F}_{n}\left(-f\right)\right]\right\}$. (16)

Thus, our task is to calculate the characteristic functions of *n*-th order ISIs of the jittered spike train. First we note that a jittered *n*-th order ISI is:

$\sim I_{n,k}=I_{n,k}+\sum_{j=0}^{n-1} \xi_{j+k}-\sum_{j=0}^{n-1} \xi_{j+k-1}= I_{n,k}+\xi_{k+n-1}-\xi_{k-1}$ .

That is, it can be represented by a sum of two independent random variables, $I_{n,k}$ and $\xi_{k+n-1}-\xi_{k-1}$. The latter variable is zero-mean with $\text{SD}=2\sigma_{\text{J}}^{2}$. Therefore the characteristic function of $\sim I _{n,k}$ is the product of characteristic function $\widehat{F}_{n}\left(f\right)$ and the square of the characteristic function of the jitter variable, $\hat{g}^{2}\left(f\right)$, $\widetilde{\widehat{F}}_{n}\left(f\right)=\hat{g}^{2}\left(f\right)\widehat{F}_{n}\left(f\right)$. Combining this last equation with Eq. 16, we arrive at the equation for the PSD of the jittered spike train, $\widetilde{G}_{rr}\left(f\right)=\left[1-\hat{g}^{2}\left(f\right)\right] \bar{r}+\hat{g}^{2}\left(f\right)G_{rr}\left(f\right)$, Eq. 8. Substitution (Methods) gives Eq. 9.

*Cross Spectral Density,* $\widetilde{G}_{sr}\left(f\right)$. The cross-spectral density in the equations for the transfer and coherence functions is defined as $G_{sr}\left(f\right)=\lim_{T\to\infty}\frac{1}{T}\left\langle\left\langle\hat{s}\left(f\right)\hat{r}*\left(f\right)\right\rangle_{r}\right\rangle_{s}$, where the hat and asterisk symbols here and in the following represent the Fourier transform and complex conjugate, respectively. The average is taken over the ensembles of spike trains and stimuli. For a spike train represented as a sum of delta functions, the Fourier transform is $\hat{r}\left(f\right)=\sum_{k} \exp\left(2\pi i f t_{k}\right)$. The Fourier transform of the jittered spike train is $\mathop{\hat{r}}\limits^{\sim} \left(f\right)=\sum_{k} \exp\left(2\pi i f t_{k}\right)\exp\left(2\pi i f \xi_{k}\right)$. Thus, in calculating the cross-spectral density of the jittered spike train, additional averaging over the jitter variable $\xi$ is also necessary. Because the jitter variable is statistically independent of both the stimulus and the spike times in the original response, the averaging of the jitter variable can be taken independently, leading to:

$\widetilde{G}_{sr}\left(f\right)=\lim_{T\to\infty}\frac{1}{T}\left\langle\left\langle\left\langle\hat{s}\left(f\right)\hat{r}*\left(f\right)\right\rangle_{r}\right\rangle_{s}\right\rangle_{\xi}\sim=\lim_{T\to\infty}\frac{1}{T}\left\langle\left\langle\hat{s}\left(f\right)\hat{r}*\left(f\right)\right\rangle_{r}\right\rangle_{s}\left\langle\exp\left(-2\pi i f\xi\right)\right\rangle_{\xi}$.

The term $\left\langle\exp\left(-2\pi i f\xi\right)\right\rangle_{\xi}$ is the complex conjugate of the characteristic function of the jitter variable, Eq. 5, leading to the final expression for the cross-spectral density of the jittered spike train, $\widetilde{G}_{sr}\left(f\right)=G_{sr}\left(f\right)\hat{g}\left(f\right)$, Eq. 10.

*Coherence Functions,* $\widetilde{C}_{\text{SR}}\left(f\right), \widetilde{C}_{\text{RR}}\left(f\right)$. The SR or RR coherence function for a jittered spike train is obtained by substitution of $\widetilde{G}_{sr}\left(f\right)$ and $\widetilde{G}_{rr}\left(f\right)$ into Eq. 2 to obtain Eq. 11 or Eq. 12.

**Supporting References**

1. Cox DR, Lewis PAW (1966) The Statistical Analysis of Series of Events. London: Methuen.

2. Holden AV (1976) Models of the Stochastic Activity of Neurones. Berlin: Springer-Verlag.
